# Prediction of short-term prognosis of patients with hypertensive intracerebral hemorrhage by radiomic-clinical nomogram

**DOI:** 10.3389/fneur.2023.1053846

**Published:** 2023-02-03

**Authors:** Jing Wang, Lu Zhou, Yuanyuan Chen, Hongli Zhou, Yuanxin Tan, Weijia Zhong, Zhiming Zhou

**Affiliations:** ^1^Department of Radiology, The Second Affiliated Hospital of Chongqing Medical University, Chongqing, China; ^2^Department of Radiology, The Third Affiliated Hospital of Chongqing Medical University, Chongqing, China; ^3^Department of Radiology, Nanchong Central Hospital, Nanchong, Sichuan, China

**Keywords:** hypertensive intracerebral hemorrhage, short-term prognosis, radiomics, nomogram, computed tomography

## Abstract

Hypertensive intracerebral hemorrhage (HICH) is the most common type of spontaneous intracerebral hemorrhage in China which is associated with high mortality and disability. We sought to develop and validate a noncontrast computed tomography (NCCT)-based nomogram model to achieve short-term prognostic prediction for patients with HICH. We retrospectively studied 292 patients with HICH from two medical centers, and they were divided into training (*n* = 151), validation (*n* = 66), and testing cohorts (*n* = 75). Based on radiomics, univariate and multivariate, and logistic regression analyses, four models (black hole sign, clinical, radiomics score, and combined models) were established to predict the prognosis of patients with HICH 30 days after the onset. The results suggested that the combined model had the best predictive performance with the area under the receiver operating characteristic curve (AUC) of 0.821, 0.816, and 0.815 in the training, validation, and testing cohorts, respectively. In addition, a radiomics-clinical (R-C) nomogram was visualized. A calibration curve analysis showed that the R-C nomogram had satisfactory calibration in the three cohorts. A decision curve analysis demonstrated that the R-C nomogram was clinically valuable. Our results suggest that the R-C nomogram can accurately and reliably predict the short-term prognosis of patients with HICH and provide a useful evaluation for making individualized treatment plans.

## Introduction

Hypertensive intracerebral hemorrhage (HICH) is defined as the sudden primary parenchymal hemorrhage in patients with clear hypertension, which occurs in the basal nucleus, the thalamus, the ventricle, the cerebellum, or the brain stem. As the most common type of spontaneous intracerebral hemorrhage (sICH), HICH accounts for ~70% of sICH with high mortality, incidence, and disability (1, 2). The 30-day mortality of HICH is as high as 45% (3). Despite the improvements in medicine in recent years, there has been no significant breakthrough in the treatment (4). Therefore, to effectively intervene HICH, identifying the prognostic risk factors early and establishing an efficient predictive model is important.

Recently, some research studies focused on the prognosis study of patients with sICH based on their clinical characteristics and radiological signs. A lower Glasgow Coma Scale (GCS) score (5) and larger hematoma volume (6) have been proven as independent risk factors for poor prognosis. Although these clinical factors are important, they do not cover all the predictive information obtained from patients. Radiological signs such as the black hole sign, the hypodensity sign, the blend sign, the swirl sign, the island sign, and the satellite sign indicate poor prognosis (7). Among them, the island sign and the satellite sign reflect the irregular shape of the hematoma and may represent multifocal small bleeding around the main hematoma, while the other signs reveal the heterogeneous density of the hematoma, indicating active bleeding. However, the identification of radiological signs is easily influenced by the subjective evaluation of doctors (8). In addition, these radiological signs, as qualitative or semiquantitative markers, have limited sensitivity and accuracy in predicting the outcomes of sICH (9).

Radiomics provides new insights into hematoma because of its ability to extract and analyze several quantitative imaging features from various types of medical images (10). Several researchers applied radiomics to predict hematoma expansion and prognosis in patients with sICH (11–14). Nevertheless, regarding HICH, the most common type of sICH, only a few studies relatively focus on it. In the present research, we designed a study to compare the performances of the black hole sign, the clinical model, the radiomics score (Rad-score), and the combined model in predicting the prognosis of patients with HICH. Then, we further attempted to establish an individualized nomogram to facilitate doctors to assess prognostic risk stratification for patients with HICH.

## Materials and methods

### Participants

All patients with a confirmed diagnosis of hypertension and intracerebral hemorrhage (ICH) from two tertiary care centers (center 1, the Second Affiliated Hospital of Chongqing Medical University and center 2, the Nanchong Central Hospital) were retrospectively reviewed between October 2014 and January 2020. Hypertension was defined as a systolic blood pressure of >140 mm Hg and/or a diastolic blood pressure of >90 mm Hg, or self-reported hypertension refers to being diagnosed by a physician and using antihypertensive medicines during the past 2 weeks. ICH was diagnosed by noncontrast computed tomography (NCCT) images, which resemble patchy high-density shadows (50–80 Hounsfield units) within the brain parenchyma. The inclusion criteria for this study were as follows: (1) the age of patients of >18 years old and (2) the time of baseline cranial NCCT of < 24 h. The exclusion criteria were as follows: (1) primary intraventricular hemorrhage or multifocal hematoma, (2) hematoma less than 1 ml in volume at the baseline, (3) secondary ICH due to head trauma, abnormal vascular structures, coagulation dysfunction, blood diseases, systemic diseases, and brain tumors, (4) severe artifacts on NCCT images, and (5) data missing. This study was approved by the ethics committee of our hospital (decision number [2019] 19) and the requirement for patient informed consent was waived.

**Figure 1:**
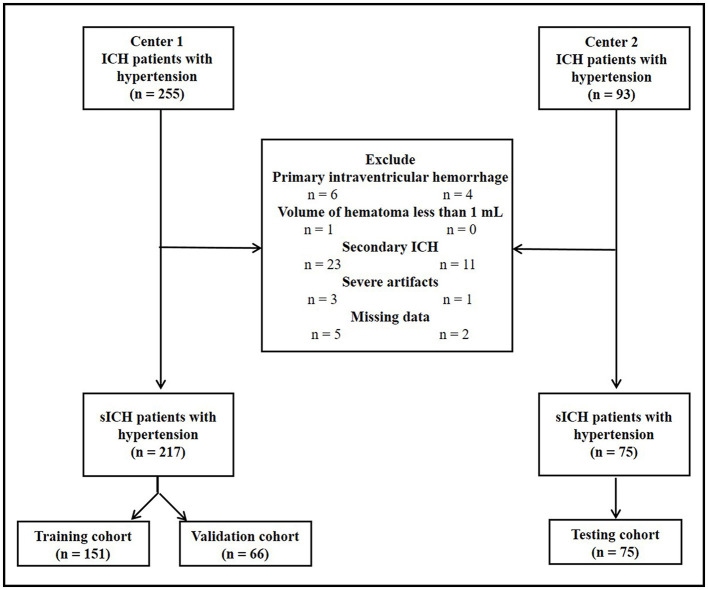
A flowchart of patient inclusion.

The flowchart of patient inclusion is shown in Figure 1. Finally, 217 consecutive patients with HICH from center 1 and 75 consecutive patients with HICH from center 2 were retrospectively reviewed. All patients in center 1 were randomly divided into training (*n* = 151, 70%) and validation cohorts (*n* = 66, 30%) by using a computer-aided algorithm. Patients from center 2 served as the testing cohort (*n* = 75).

### Clinical and imaging characteristics and conventional prognosis assessment

The demographic characteristics, the results of laboratory examinations, and NCCT imaging characteristics of the three cohorts were collected from the Hospital Information System and the Picture Archiving and Communication System of the two centers and are summarized in Supplementary material (Supplementary Table S1). The black hole sign was defined as a hypodense area completely encapsulated by the hyperattenuating hematoma with ≥28 Hounsfield unit difference between the two regions (15). On NCCT, the midline shift, intraventricular hemorrhage, subarachnoid hemorrhage, and black hole sign were blindly assessed by three experienced radiologists. If there was disagreement among them, these characteristics would be evaluated after consultation.

At 30 days after the onset, the prognosis of patients with HICH was evaluated by the modified Rankin Scale (mRS) scores, with scores 0–3 representing a good outcome and 4–6 representing a poor outcome (16).

### Image acquisition

Three different CT scanners (Aquilion ONE, Canon Medical Systems, Otawara, Japan; Ingenuity, Philips Healthcare, Best, Netherlands; and Lightspeed VCT, GE Medical Systems, Waukesha, WI, USA) were used to obtain all NCCT images. The standard head CT scanning protocol was as follows: a tube voltage of 120 kV, a tube current of 250–300 mA, a matrix size of 512 × 512, gantry rotation of 0.4–0.6 s, a field of view of 25 cm, a slice thickness of 1 mm, and a detector width of 0.5 mm or 0.625 mm. The scanning range was from the skull base to the cranium.

### Radiomics analysis

The radiomics analysis in this study was carried out according to the standard pipeline (17). The pipeline for the radiomics analysis is as follows: (1) image preprocessing, (2) lesion segmentation, (3) feature extraction, (4) feature harmonization, (5) features selection, and (6) model construction and evaluation.

First, image normalization was performed to reduce the possible impact of different scanning devices on the results, which is detailed in the Supplementary material. Second, an experienced radiologist (ZMZ, 8 years of service) manually drew the edge of the hematoma layer by layer on an axial image of NCCT by using the Artificial Intelligence Kit (AK) software (version 3.4.1, GE Healthcare). Then, the volume of interest (VOI) was generated by using the AK software and the volume of the hematoma was calculated automatically by summing the number of voxels of the VOI. Using AK software, a total of 396 radiomic features were extracted automatically from VOI. All radiomic features were categorized into five types: (1) morphological features (*n* = 9); (2) texture features based on other methods (*n* = 54); (3) histogram features (*n* = 42); (4) gray-level cooccurrence matrix (GLCM) features (*n* = 100); and (5) run-length matrix (RLM) features (*n* = 191). The interclass correlation coefficient (ICC) was employed to evaluate the stability of the radiomic features. The reproducibility analysis is also detailed in the Supplementary material.

**Figure 2:**
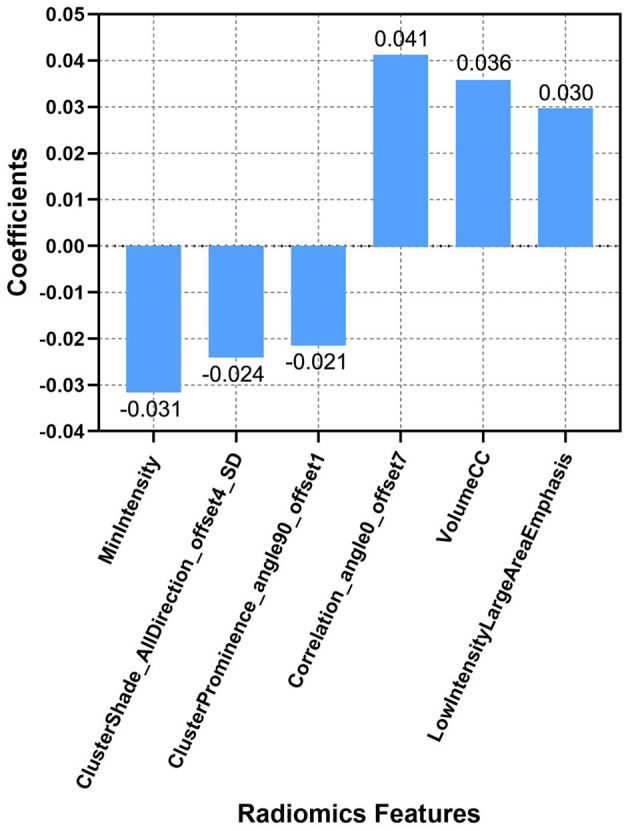
Descriptions of the six optimal radiomic features used to calculate the Rad-score. The *x*-axis represents the individual radiomic features, with their coefficients in the LASSO regression analysis plotted on the *y*-axis.

First, radiomic feature harmonization was performed as detailed in the Supplementary material. Radiomic feature selection was done using the Python software (version 3.2). Specifically, 24 radiomic features were first selected by using the Student's *t-*test. After using the least absolute shrinkage and selection operator (LASSO) and 10-fold cross-validation, only six optimal radiomic features (cluster prominence feature, cluster shade feature, MinIntensity feature, correlation feature, volumeCC feature, and the low-intensity large area emphasis feature) remained (Figure 2).

The Rad-score of each patient with HICH was calculated according to the six optimal features: Rad-score = ($\sum \beta_{\text{i}}^{*}$X_i_ + Intercept); in the formula, X_i_ represents the *i*-th selected feature, β_i_ was its coefficient, and intercept = 0.403. The radiomic score based on the NCCT images is described in Supplementary Figure S1 of the Supplementary material.

### Model construction and evaluation

First, the risk factors of the clinical and radiological variables were screened by univariate analysis; a *P*-value of < 0.1 was considered to be statistically significant. Then, the above significant risk factors were included in a multivariate analysis to determine the independent risk factors; a *P*-value of < 0.05 was considered to be statistically significant. The independent risk factors of the clinical and radiological variables were input into the logistic regression model (enter method) to construct the clinical model. The clinical model and the Rad-score model were employed together to build the combined model.

All models were verified separately in the validation cohort and testing cohort. The area under the receiver operating characteristic curve (AUC) was calculated to evaluate the discriminative ability of each model in the three cohorts.

A nomogram was constructed based on the optimal prediction model. The DeLong test was applied to compare the AUC of the nomogram among the three cohorts. A calibration curve was employed to graphically evaluate the calibration capability of the nomogram in each cohort, which described the degree of fit between the actual and nomogram-predicted prognosis of patients with HICH at 30 days. A decision curve analysis (DCA) was performed to assess the clinical application benefits of the nomogram.

### Statistical analysis

Statistical analysis was performed using the R software (version 3.6.0; http://www.rproject.org/). Continuous variables were summarized as means ± standard deviation or as medians and interquartile ranges as appropriate, and categorical variables were presented using counts (percentages). The normality of continuous variables was evaluated by the Kolmogorov–Smirnov test. Independent sample *t-*test or Mann–Whitney U-test was used for continuous variables. The chi-square test or Fisher's exact test was applied to compare categorical variables.

## Results

### Baseline characteristics

In the training cohort, 61 out of the 151 (40.4%) had poor outcomes (mRS > 3 at 30 days). Between good outcomes and poor outcomes, there were significant differences in age (*P* = 0.063), GCS score (*P* < 0.001), serum glucose (*P* = 0.003), creatinine (*P* = 0.070), baseline HICH volume (*P* = 0.044), midline shift (*P* = 0.001), black hole sign (*P* < 0.001), and Rad-score (*P* < 0.001) (Table 1). The multivariate analysis revealed the following independent predictors of the 30-day poor outcomes at baseline (Table 2): GCS score (odds ratio [OR] = 0.729, 95% confidence interval [CI]: 0.615–0.865, and *P* < 0.001), midline shift (OR = 2.864, 95%CI: 1.206–6.799, and *P* = 0.017), and black hole sign (OR = 3.304, 95%CI: 1.313–8.311, and *P* = 0.011). Although we did not find significance in relation to hematoma volume, the most important factor for determining the prognosis of patients with HICH (18), we still included it to construct the clinical model at the baseline.

**Table 1:** Comparison of baseline characteristics between patients with good outcomes and those with poor outcomes in the training cohort.

**Variables**	**Poor outcome** **(*n =* 61)**	**Good outcome** **(*n =* 90)**	***P-*value**
Age (y)	62.92 ± 13.84	58.73 ± 13.22	0.063
Men	35 (57.4%)	59 (65.6%)	0.309
Diabetes mellitus	11 (18.0%)	10 (11.1%)	0.228
Admission SBP (mmHg)	182.57 ± 34.01	175.97 ± 26.64	0.184
Admission DBP (mmHg)	101.44 ± 20.69	102.72 ± 17.06	0.679
Onset-to-CT time (h)	2.00 [1.25–3.50]	3.00 [1.38–4.00]	0.163
GCS score	11.00 [9.00–14.00]	13.00 [12.00–14.00]	< 0.001
WBC (10^9^/L)	9.05 [7.15–11.06]	8.53 [7.07–10.49]	0.339
Hemoglobin (g/L)	135.70 ± 19.44	141.56 ± 24.98	0.126
Platelets (10^9^/L)	179.90 ± 74.64	197.87 ± 57.47	0.115
APTT (s)	33.20 [31.25–35.25]	34.40 [31.28–38.13]	0.135
INR	1.03 ± 0.11	1.03 ± 0.09	0.746
Fibrinogen (g/L)	3.08 ± 0.82	3.21 ± 1.02	0.433
Serum glucose (mmol/L)	7.88 [6.13–9.11]	6.14 [5.43–8.27]	0.003
Serum Mg(mmol/L)	0.82 ± 0.11	0.82 ± 0.10	0.929
Serum Ca(mmol/L)	2.21 ± 0.15	2.24 ± 0.19	0.299
Serum Na(mmol/L)	139.54 ± 5.10	139.76 ± 3.65	0.778
Creatinine(umol/L)	56.50 [46.95–74.90]	65.25 [52.35–82.75]	0.070
Urea(mmol/L)	5.96 [4.36–7.49]	5.62 [4.36–6.80]	0.445
Uric acid(umol/L)	296.87 ± 112.85	328.55 ± 119.15	0.104
HDL-C (mmol/L)	1.12 [0.96–1.37]	1.11 [0.90–1.28]	0.405
LDL-C (mmol/L)	2.42 ± 0.96	2.54 ± 0.82	0.418
Serum albumin (g/L)	40.79 ± 5.00	42.04 ± 5.22	0.144
ApoA-I (g/L)	1.54 ± 0.31	1.50 ± 0.34	0.468
ApoE (mg/L)	36.73 ± 13.81	35.44 ± 9.46	0.526
Baseline HICH volume (ml)	17.63 [8.31–22.61]	11.60 [6.97–18.90]	0.044
Midline shift	31 (50.8%)	23 (25.6%)	0.001
IVH extension	19 (31.1%)	22 (24.4%)	0.363
SAH	3 (4.9%)	1 (1.1%)	0.303
Black hole sign	24 (39.3%)	13 (14.4%)	< 0.001
Rad-score	0.45 ± 0.12	0.38 ± 0.10	< 0.001
Surgical intervention	17 (27.9%)	22 (24.4%)	0.637
30-day mRS	4.00 [4.00–5.00]	3.00 [2.00–3.00]	< 0.001

Data are presented as mean and standard deviation, median and interquartile ranges, or numbers and percentages in parenthesis. SBP, systolic blood pressure; DBP, diastolic blood pressure; GCS, Glasgow Coma Scale; WBC, white blood cell count; APTT, activated partial thromboplastin time; INR, international normalized ratio; Mg, magnesium; Ca, calcium; Na, sodium; HDL-C, high-density lipoprotein cholesterol; LDL-C, low-density lipoprotein cholesterol; ApoA-I, apolipoprotein A-I; ApoE, apolipoprotein E; HICH, hypertensive intracerebral hemorrhage; IVH, intraventricular hemorrhage; SAH, subarachnoid hemorrhage; Rad-score, radiomics score; mRS, modified Rankin Scale.

**Table 2:** Multivariate analysis of baseline characteristics of poor outcomes in the training cohort.

**Variable**	**OR**	**95%CI**	***P-*value**
Age (y)	1.025	0.994–1.057	0.121
GCS score	0.729	0.615–0.865	< 0.001
Creatinine	1.001	0.998–1.004	0.336
Serum glucose (mmol/L)	1.063	0.923–1.225	0.394
Baseline HICH volume (ml)	0.983	0.951–1.017	0.327
Midline shift	2.864	1.206–6.799	0.017
Black hole sign	3.304	1.313–8.311	0.011

OR, indicates odds ratio; CI, confidence interval; GCS, Glasgow Coma Scale; HICH, hypertensive intracerebral hemorrhage.

Finally, the variables of the combined model: GCS score (odds ratio [OR] = 0.730, 95% confidence interval [CI]: 0.617–0.864, and *P* < 0.001), hematoma volume (OR = 0.984, 95%CI: 0.950–1.020, and *P* = 0.381), midline shift (OR = 2.366, 95%CI: 0.988–5.669, and *P* = 0.053), black hole sign (OR = 3.897, 95%CI: 1.530–9.926, and *P* = 0.004), and Rad-score (OR = 289.438, 95%CI: 6.132–13,662.583, and *P* = 0.004).

### Model evaluation

**Figure 3:**
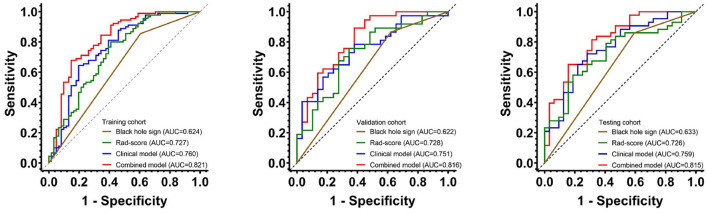
The receiver operating characteristic curves of the black hole sign, Rad-score model, clinical model, and combined model for assessing the 30-day clinical functional outcomes in the training, validation, and testing cohorts.

**Figure 4:**
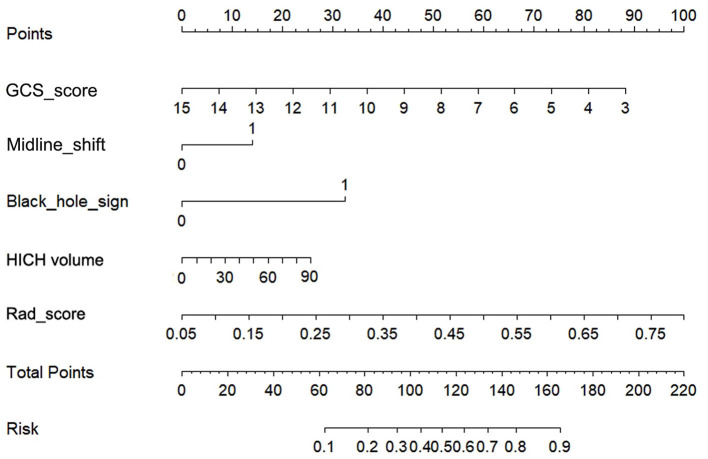
The radiomics-clinical nomogram for assessing the 30-day clinical functional outcomes. Five variables, two points, and a risk estimation were incorporated into the nomogram. To use this nomogram, we added the points corresponding to each variable to get the total points and then find the corresponding risk estimation.

The AUCs of black hole sign, Rad-score, clinical, and combined models in the training, validation, and testing cohorts were (0.624, 0.727, 0.760, and 0.821), (0.622, 0.728, 0.751, and 0.816), and (0.633, 0.726, 0.759, and 0.815), respectively (Figure 3). The combined model was verified as the optimal model with the highest AUC among the three cohorts. Based on the combined model, a visualized radiomic-clinical (R-C) nomogram was established (Figure 4). The DeLong test showed no significant difference in the AUC of the R-C nomogram between the training and validation cohorts (*P* = 0.491), between the training and testing cohorts (*P* = 0.250), and between the testing and validation cohorts (*P* = 0.697).

**Figure 5:**
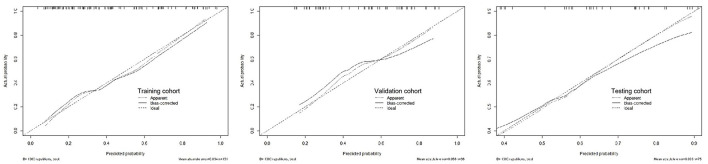
The calibration curves for the radiomics-clinical nomogram in the training, validation, and testing cohorts.

The calibration curve of the R-C nomogram showed favorable agreement among the three cohorts with regard to the predicted and observed probabilities of high-risk 30-day poor outcomes (Figure 5). The Hosmer–Lemeshow test showed no statistical significance in the training (*P* = 0.155), validation (*P* = 0.089), and testing cohorts (*P* = 0.067).

**Figure 6:**
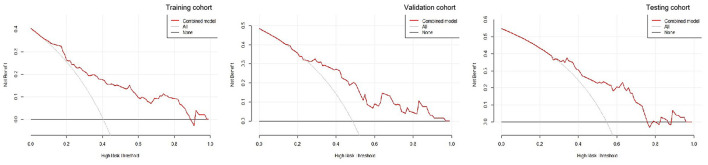
The decision curves for the radiomics-clinical nomogram in the training, validation, and testing cohorts.

A decision curve analysis revealed that the R-C nomogram for predicting the 30-day prognosis provided greater net benefit than the treat-all-patients and the treat-none-patients in the training, validation, and testing cohorts, which suggested the clinical value of the R-C nomogram (Figure 6).

## Discussion

Recently, although some studies have established different prognostic prediction models on patients with sICH, there are relatively few studies that focus on patients with HICH alone (19, 20). Most of them only include radiomic features or clinical factors and lack external data validation. Focusing on HICH, we included patients with HICH from two centers and constructed four models with more data to verify their 30-day outcome predictive power.

A noncontrast computed tomography is the first-line imaging method for acute ICH in the world, which can be used to reflect the heterogeneity and morphological characteristics of hematoma. Some NCCT-based radiological signs have been employed to predict the prognosis of patients with sICH (7). In our study, the black hole sign showed the lowest performance in contrast to the other three models (AUCs of 0.624 in the training cohort, 0.622 in the validation cohort, and 0.633 in the testing cohort). This is in line with Li et al.'s results that showed the low sensitivity of the black hole sign (21).

Risk factors, including the GCS score, hematoma volume, age, and intraventricular hemorrhage (IVH), have been proven to be predictors of the prognosis for patients with sICH (5, 6, 18, 22–26). Based on these risk factors, many clinical scores have been developed such as the ICH score (27), the FUNC score (Functional Score) (23), and the ICH-FOS score (ICH Functional Outcome Score) (28). Nevertheless, these clinical scores only focused on the clinical aspects and basic hematoma features and they have relatively low accuracy. In these clinical scores, the hematoma volume evaluated by the Tada formula is proved to be inaccurate, especially for irregular hematomas (29). In our research, we used the AK software to semiautomatically calculate hematoma volume and improve accuracy. In addition, to our knowledge, different treatment methods will affect the prognosis of patients with HICH. However, in our study, there was no statistical difference between the treatment methods and the prognosis; we speculated that it might be caused by the relatively small sample of patients with HICH.

Radiomics automatically extracts a large number of high-throughput and quantitative features from the medical images, which include first-, second-, and higher-order statistics (30). It can find features invisible under the naked eye in contrast to the traditional practice of regarding medical images as pictures for visual interpretation only. Our Rad-score model successfully predicted the 30-day outcomes of patients with HICH. However, the predictive power (AUCs of 0.727, 0.728, and 0.726 in the training, validation, and testing cohorts) was slightly lower than that of the clinical model (AUCs of 0.760, 0.751, and 0.759 in the training, validation, and testing cohorts). The study by Pszczolkowski et al. demonstrates a similar result to ours, showing the lower predictive performance of the radiomics model compared to the clinical model (11). In addition, Wu et al. found that more combinations of different types of data would increase the performance of the prediction of prognosis for patients with HICH (19). Our clinical model covered the clinical factors and the black hole sign, which might increase the predictive power.

Previous studies showed that the R-C combined model usually has a higher prediction ability. Chen et al. found that, compared with the clinical or radiomics model, the combined model has the highest sensitivity and AUC in predicting hematoma expansion in sICH patients (31). In addition, in a study about predicting the outcomes of acute ischemic stroke at 6 months after hospital discharge, the AUC of the R-C combined model is 0.868 in the training cohort and 0.890 in the validation cohort, which is significantly higher than that of the clinical or radiomics model (32). Similarly, a machine learning model based on PET/CT radiomics and clinical characteristics predicted the tumor immune microenvironment profiles of nonsmall cell lung cancer, which showed that the R-C combined model has the best performance (33). In our research, the GCS, hematoma volume, midline shift, black hole sign, and Rad-score were employed to build the R-C combined model for predicting the short-term prognosis of HICH patients. It was verified to have the best predictive performance (AUCs of 0.821, 0.816, and 0.815 in the training, testing, and validation cohorts) compared with our other three models. At the same time, the R-C nomogram constructed based on the combined model has been proven to be in good agreement with the actual clinical outcomes by the calibration curves of the three cohorts. It could provide a personalized 30-day prediction for patients with HICH.

However, this study had some limitations. First, although image normalization was performed prior to image analysis, the impact of imaging data from different CT scanners on the performance of the prediction models remained unclear. Second, among the numerous radiological signs, only the most representative black hole sign in terms of density heterogeneity was employed in this study (7), and hence, other radiological signs need to be examined in the future. In addition, although all patients with HICH were treated according to the Chinese guidelines for diagnosis and treatment of acute intracerebral hemorrhage, different treatment measures received by patients may confound the factors for predicting prognosis. Finally, this study was a retrospective study, and prospective studies with a higher number of patients need to be conducted in the future.

## Conclusion

The combined model consisting of the black hole sign, GCS score, midline shift, hematoma volume, and Rad-score can predict the short-term prognosis of patients with HICH early, easily, and accurately. After visualizing the model as an R-C nomogram, it is more convenient for clinicians to make correct clinical decisions and devise the best treatment plan.

## Data availability statement

The raw data supporting the conclusions of this article will be made available by the authors, without undue reservation.

## Ethics statement

The studies involving human participants were reviewed and approved by the Ethics Committee of the Second Affiliated Hospital of Chongqing Medical University (No. [2019] 19). The Ethics Committee waived the requirement of written informed consent for participation.

## Author contributions

WZ and ZZ: conception, design, analysis, interpretation of data, critical revision of manuscript for intellectual content, study supervision, technical and administrative supports, and approving the final version of the manuscript on behalf of all authors. JW, LZ, YC, HZ, and YT: acquisition of data. JW and ZZ: data processing and statistical analysis. JW and LZ: writing the first draft of the manuscript. All authors contributed to the article and approved the submitted version.
