# Supplementary material for: Prediction of short-term prognosis of patients with hypertensive intracerebral hemorrhage by radiomic-clinical nomogram
Source: Front Neurol. 2023 Feb 3;14:1053846. doi: 10.3389/fneur.2023.1053846 (PMC9935706; doi:10.3389/fneur.2023.1053846)
Supplement: Supplementary file 1 [file Data_Sheet_1.docx]

**MATERIALS AND METHODS**

**Image normalization**

Image normalization was performed as follows: (1) image registration: every noncontrast computed tomography (NCCT) image slice from the raw data was resampled to a unified pixel dimension size of 1.0×1.0×1.0 mm^3^; (2) gray level discretization: image intensity of every NCCT image were normalized by the gray level discretization method with a fixed number of bins (256 bins); (3) NCCT images were viewed in a fixed head window (level = 50 Hounsfield unit; width = 110 Hounsfield unit).

**Reproducibility analysis**

Inter-observer and intra-observer reproducibility analyses were performed on 50 patients randomly selected from the training cohort. The region of interest (ROI) of each patient was manually segmented again by the same experienced radiologist after 2 weeks interval and by another experienced radiologist using the same method. After extracting radiomics features, the intraclass correlation coefficient (ICC) was calculated to assess the reproducibility of radiomics features. The results showed that 364 (91.9%) radiomics features showed ICC > 0.8 in the inter-observer reproducibility analysis and 370 (93.4%) radiomics features showed ICC > 0.8 in the intra-observer reproducibility analysis.

**Radiomics Features Harmonization**

Before radiomics features selection, harmonization in the feature domain was performed. First, variables with zero variance were excluded from analyses, and the missing values were replaced by the median. Then the data were standardized by using zero-mean normalization.

**Table S1 Clinical and imaging characteristics of the three cohorts.**

| Variables | Training Cohort  (n = 151) | Validation Cohort  (n = 66) | Testing Cohort  (n = 75) | *P* value |
| --- | --- | --- | --- | --- |
| Age (y) | 60.42 ± 13.58 | 63.06 ± 12.00 | 66.40 ± 12.53 | 0.005 |
| Male | 94 (62.3%) | 41 (62.1%) | 49 (65.3%) | 0.890 |
| Diabetes mellitus | 21 (13.9%) | 10 (15.2%) | 7 (9.3%) | 0.530 |
| Admission SBP (mmHg) | 178.64 ± 29.91 | 177.92 ± 25.56 | 174.68 ± 22.63 | 0.583 |
| Admission DBP (mmHg) | 102.21 ± 18.55 | 100.03 ± 13.68 | 98.24 ± 15.66 | 0.235 |
| Onset-to-CT time (h) | 2.00 [1.50-4.00] | 3.00 [2.00-4.00] | 3.00 [2.00-7.00] | <0.001 |
| GCS score | 13．00 [10.00-14.00] | 13.00 [10.00-14.00] | 13.00 [10.00-14.00] | 0.750 |
| WBC (10^9^/L) | 8.76 [7.14-10.68] | 7.77 [5.98-10.80] | 8.80 [6.57-11.50] | 0.244 |
| Hemoglobin (g/L) | 139.19 ± 23.01 | 135.30 ± 20.85 | 134.89 ± 17.96 | 0.259 |
| Platelets (10^9^/L) | 190.87 ± 64.81 | 184.12 ± 71.94 | 174.32 ± 58.27 | 0.196 |
| APTT (s) | 33.90 [31.30-36.30] | 33.65 [31.48-36.80] | 24.60 [21.60-28.20] | <0.001 |
| INR | 1.03 ± 0.10 | 1.03 ± 0.16 | 1.01 ± 0.11 | 0.504 |
| Fibrinogen (g/L) | 3.16 ± 0.94 | 3.03 ± 0.78 | 2.44 ± 0.59 | <0.001 |
| Serum glucose (mmol/L) | 6.77 [5.66-8.76] | 6.65 [5.75-8.36] | 7.32 [6.22-9.50] | 0.100 |
| Serum Mg (mmol/L) | 0.82 ± 0.11 | 0.79 ± 0.07 | 0.83 ± 0.11 | 0.045 |
| Serum Ca (mmol/L) | 2.23 ± 0.18 | 2.20 ± 0.12 | 2.27 ± 0.13 | 0.027 |
| Serum Na (mmol/L) | 139.67 ± 4.28 | 140.06 ± 4.48 | 141.77 ± 4.55 | 0.003 |
| Creatinine (umol/L) | 63.00 [49.30-79.70] | 56.30 [44.00-69.25] | 63.50 [50.30-73.50] | 0.087 |
| Urea (mmol/L) | 5.71 [4.37-7.15] | 5.51 [4.50-6.46] | 5.10 [3.90-6.20] | 0.031 |
| Uric acid (umol/L) | 315.75 ± 117.31 | 274.53 ± 98.30 | 314.01 ± 128.69 | 0.046 |
| HDL-C (mmol/L) | 1.11 [0.93-1.30] | 1.29 [1.05-1.49] | 1.45 [1.19-1.69] | <0.001 |
| LDL-C (mmol/L) | 2.49 ± 0.88 | 2.53 ± 0.94 | 2.52 ± 0.68 | 0.949 |
| Serum albumin (g/L) | 41.54 ± 5.15 | 40.21 ± 7.78 | 42.31 ± 2.82 | 0.071 |
| ApoA-I (g/L) | 1.51 ± 0.32 | 1.61 ± 0.36 | 1.22 ± 0.28 | <0.001 |
| ApoE (mg/L) | 35.96 ± 11.39 | 33.46 ± 9.82 | 36.52 ± 12.60 | 0.228 |
| Baseline HICH volume (ml) | 14.09 [7.53-22.04] | 12.83 [6.30-18.46] | 11.79 [5.40-21.03] | 0.297 |
| Midline shift | 54 (35.8%) | 24 (36.4%) | 32 (42.7%) | 0.583 |
| IVH extension | 41 (27.2%) | 17 (25.8%) | 23 (30.7%) | 0.788 |
| SAH | 4 (2.6%) | 1 (1.5%) | 5 (6.7%) | 0.184 |
| Black hole sign | 37 (24.5%) | 16 (24.2%) | 19 (25.3%) | 0.987 |
| Rad-score | 0.41 ± 0.11 | 0.41 ± 0.14 | 0.41 ± 0.13 | 0.971 |
| Surgical intervention | 39 (25.8%) | 16 (24.2%) | 18 (24.0%) | 0.944 |
| 30-day mRS | 3.00 [3.00-4.00] | 3.00 [3.00-4.00] | 4.00 [3.00-5.00] | 0.520 |
| Poor outcome | 61 (40.4%) | 28 (42.4%) | 41 (54.7%) | 0.118 |

Data are presented as mean and standard deviation, median and interquartile ranges, or numbers and percentages in parenthesis. SBP: systolic blood pressure; DBP: diastolic blood pressure; GCS: Glasgow Coma Scale; WBC: white blood cell count; APTT: activated partial thromboplastin time; INR: international normalized ratio; Mg: magnesium,; Ca: calcium; Na: sodium; HDL-C: high density lipoprotein cholesterol; LDL-C: low density lipoprotein cholesterol; ApoA-I: apolipoprotein A-I; ApoE: apolipoprotein E; HICH: hypertensive intracerebral hemorrhage; IVH: intraventricular hemorrhage; SAH: subarachnoid hemorrhage; Rad-score: radiomics score; mRS: modified Rankin Scale.


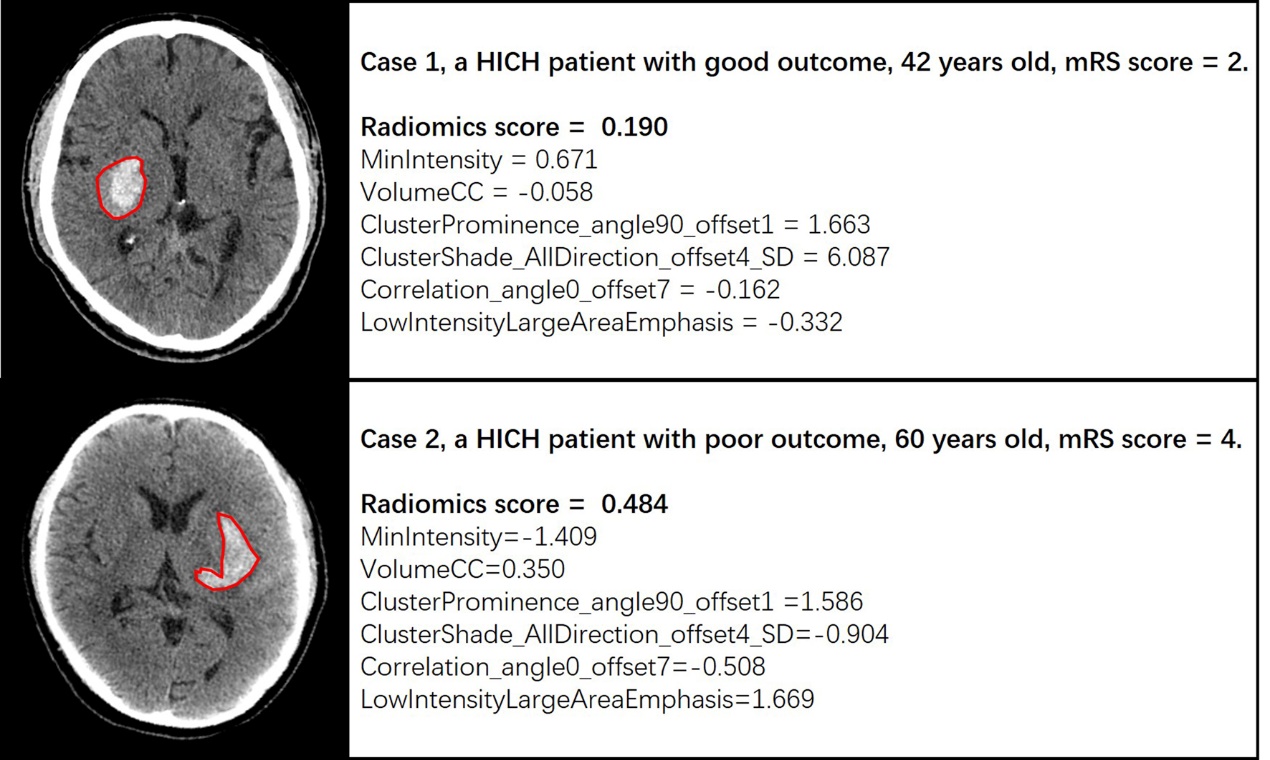


Figure S1 | The radiomics features and radiomics score based on NCCT images from two cases with HICH.
